# Long-haul COVID: healthcare utilization and medical expenditures 6 months post-diagnosis

**DOI:** 10.1186/s12913-022-08387-3

**Published:** 2022-08-08

**Authors:** Antonios M. Koumpias, David Schwartzman, Owen Fleming

**Affiliations:** 1 Department of Social Sciences, University of Michigan-Dearborn, Dearborn, USA; 2 Olin College of Business, Washington University in St. Louis, St. Louis, USA; 3 Department of Economics, Wayne State University, 656 W. Kirby St FAB 2140, Detroit, USA

**Keywords:** Long-haul COVID-19, Healthcare Utilization, Medical Expenditures

## Abstract

**Background:**

Despite extensive evidence that COVID-19 symptoms may persist for up to a year, their long-term implications for healthcare utilization and costs 6 months post-diagnosis remain relatively unexplored. We examine patient-level association of COVID-19 diagnosis association of COVID-19 diagnosis with average monthly healthcare utilization and medical expenditures for up to 6 months, explore heterogeneity across age groups and determine for how many months post-diagnosis healthcare utilization and costs of COVID-19 patients persist above pre-diagnosis levels.

**Methods:**

This population-based retrospective cohort study followed COVID-19 patients’ healthcare utilization and costs from January 2019 through March 2021 using claims data provided by the COVID-19 Research Database. The patient population includes 250,514 individuals infected with COVID-19 during March-September 2020 and whose last recorded claim was not hospitalization with severe symptoms. We measure the monthly number and costs of total visits and by telemedicine, preventive, urgent care, emergency, immunization, cardiology, inpatient or surgical services and established patient or new patient visits.

**Results:**

The mean (SD) total number of monthly visits and costs pre-diagnosis were .4783 (4.0839) and 128.06 (1182.78) dollars compared with 1.2078 (8.4962) visits and 351.67 (2473.63) dollars post-diagnosis. COVID-19 diagnosis associated with .7269 (95% CI, 0.7088 to 0.7449 visits; *P* < .001) more total healthcare visits and an additional $223.60 (95% CI, 218.34 to 228.85; *P* < .001) in monthly costs. Excess monthly utilization and costs for individuals 17 years old and under subside after 5 months to .070 visits and $2.77, persist at substantial levels for all other groups and most pronounced among individuals age 45–64 (.207 visits and $73.43) and 65 years or older (.133 visits and $60.49).

**Conclusions:**

This study found that COVID-19 diagnosis was associated with increased healthcare utilization and costs over a six-month post-diagnosis period. These findings imply a prolonged burden to the US healthcare system from medical encounters of COVID-19 patients and increased spending.

**Supplementary Information:**

The online version contains supplementary material available at 10.1186/s12913-022-08387-3.

## Background

COVID-19 caused by the novel coronavirus SARS-CoV-2 can increase healthcare utilization and costs directly in response to COVID-19 related illness and indirectly to treat non COVID-19 related health conditions identified in the course of the COVID-19 medical encounter or decrease utilization and costs due to deferral of care [1–, 2–8]. If the post-acute sequelae of COVID-19 symptoms (long-haul COVID) are not ephemeral, the prolonged burden they place on the healthcare system could further undermine its capacity to cater to the growing healthcare needs of the population and exacerbate wasteful spending [9].

There is extensive evidence that COVID-19 leads to functional decline and that symptoms related to either mild or critical COVID-19 hospitalizations can persist for 60 days or longer [10–, 11–16]. A number of studies report symptom persistence for COVID-19 patients up to 12 months following infection [17–19]. A survey of 11–17 year-olds infected with COVID-19 revealed that symptoms persist at least 15 weeks following infection [20]. Al-Aly et al. report an increased likelihood of health resource usage by COVID-19 survivors 30 days post-discharge. [12] Chopra et al. [21] provide evidence of increased morbidity, physical and emotional symptoms among COVID-19 patients 60 days post-discharge and report that the majority of the surveyed population experienced some degree of financial impact from hospitalization. Salerno et al. find increased healthcare resource utilization by younger, non-Black COVID-19 patients and increased rates of hospitalization and mortality among older, male or Black patients. [22]

An observational cohort study of COVID-19 patients for six months post-diagnosis finds the highest number of visits during the first 30 days and a gradual decrease over time. Older age, female gender and higher BMI were associated with higher total utilization for hospitalized patients while these factors as well as non-white race/ethnicity, former smoking, and greater number of pre-existing comorbidities were all associated with increased utilization among non-hospitalized patients [23]. Chua et al. (2021) estimate mean total out-of-pocket spending and spending for facility services for COVID-19 hospitalizations in 2020 to be $788 and $3,840 for privately insured patients and $277 and $1,536 for Medicare Advantage patients. [24]. A cross-sectional cost-description study of patients in Iran hospitalized for 4 months with COVID-19 assesses the economic burden from direct medical costs at $3,755, of which $2,168 are attributed to inpatient and intensive care services [25].

Extending our understanding of the association of long-haul COVID-19 with healthcare utilization with longer-term estimates is of major public health importance, as COVID-19 has affected a sizable fraction of the population whose future medical needs and their financial implications are not well-understood [26]. Moreover, the introduction of the COVID-19 Uninsured Program by the Health Resources and Services Administration (HRSA) in response to the public health emergency offers a rare view of healthcare use and cost of a relatively understudied population, the uninsured, using claims data which offer a more holistic view of the financial records of medical encounters than electronic medical records. This study had two primary aims. First, we aimed to chart health care utilization and medical expenditures for six months following COVID-19 diagnosis among privately, publicly insured and uninsured patients in the U.S. between February 2020 and October 2021 and assess differences by age groups or by service category. Furthermore, we sought to determine for how many months post-diagnosis healthcare utilization and costs of COVID-19 patients of different age groups persist above pre-diagnosis levels.

## Methods

### Data description and sampling process

**Fig. 1:**
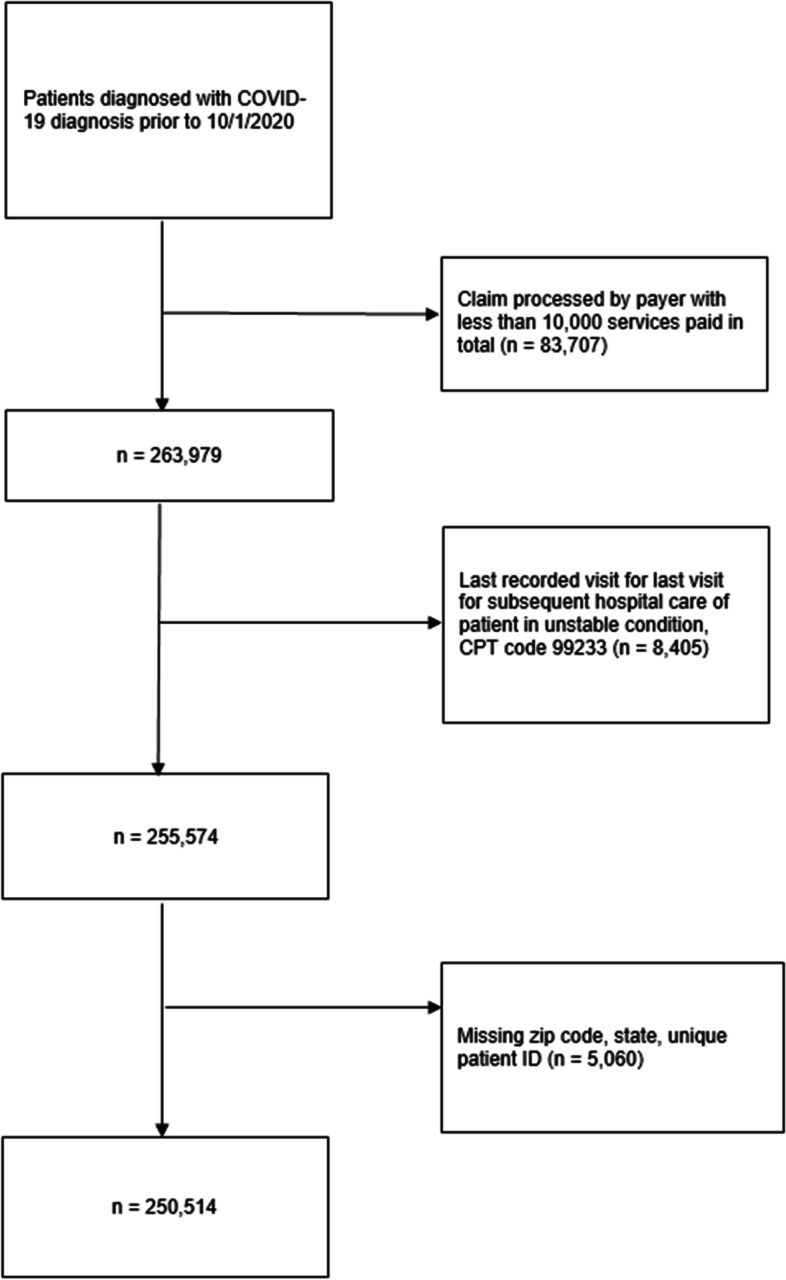
Analytic cohort construction of patients with lab-confirmed COVID-19 diagnosis. Figure 1 shows the inclusion and exclusion criteria used to construct the sample and the number of excluded observations at each step as well as the final number of the patients included in the study sample

. The dataset contains de-identified insurance claims data from providers or billers totaling 3.4 billion claims on 100 million unique patients over the last 7 years. All 50 U.S. states are represented in the patient population, albeit there is a significantly higher density of claims primarily in California and of Medicaid in Colorado, North Carolina, Texas and Washington. Our sample includes information on healthcare utilization and medical expenditures from January 1, 2019 through March 31, 2021 of patients with a lab-confirmed COVID-19 diagnosis from the beginning of the public health emergency in February 2020 through October 1, 2020. We first exclude medical claims processed by small market-size payers, defined as payers with less than 10,000 processed claims in total. This process retains over 90% of the original sample and includes claims by 169 large payers such as commercial providers, state Medicare and Medicaid programs and uninsured to minimize measurement error in payer status assignment. Next, we drop patients whose last recorded visit regarded subsequent hospital care for unstable condition or significant new complications or problems [CPT code 99,233 “Subsequent Hospital Care Evaluation and Management (E/M) Services”] to minimize concerns about sample attrition from mortality as the latter information is not available in the dataset. Finally, we exclude individuals with claims with missing zip code, state information or multiple patient IDs. These inclusion and exclusion criteria, which are presented in Fig. 1, give rise to a study sample of 3,006,168 medical claims which we collapse into a twelve-month panel of 250,514 patients whose history of medical encounters is available for six pre- and post-COVID-19 diagnosis months.

We identified different types of outpatient and inpatient medical encounters based on current procedural terminology (CPT) codes and designated broader classifications to each service, creating 10 distinct and mutually exclusive categories using a standard method of classifying services by the Health Care Cost Institute. These categories were selected to reflect responses to COVID-19 diagnosis in the modality (telemedicine), severity (immunization, cardiology, inpatient, surgery) point of care (preventive, urgent care, emergency), and patient-physician relationship (established patient visit, new patient visit). Finally, we summed all ten categories to obtain an aggregate measure of healthcare utilization and medical expenditures for every patient in each month. Pulmonary medical encounters are not used in the analysis to prevent respiratory-related medical encounters from dominating the aggregate results and highlight any smaller but meaningful and potentially unanticipated associations of COVID-19 diagnosis with a wide variety of other, non-respiratory specialty-specific healthcare utilization and costs.

### Statistical analysis

First, we compare utilization of health care services and healthcare expenditures before and after COVID-19 diagnosis using a linear regression model that includes an indicator variable that is equal to one in all post-diagnosis months; zero, otherwise, individual-level demographic characteristics and insurance status information as well as 3-digit zip code-level socio-economic factors. Specifically, we control for individual-level gender (male indicator), age organized in four groups (18–44, 45–64, > 65 years of age with patients younger than 18 years old serving as the baseline category), insurance status (Medicare, Medicaid, COVID-19 Uninsured Program by the HRSA for the uninsured and commercial private insurance as the reference category) and 3-digit zip-code level total population, percent of the population living in rural areas, percent non-white population, percent greater than 65 years of age, percent less than 18 years of age, percent unemployed, percent female, poverty rate and per capita income. We specify state fixed effects and indicators of the COVID-19 diagnosis month of the year to capture month-specific trends in the evolution of the pandemic. Standard errors are clustered at the individual level [see Additional file 1].

Next, we investigate the dynamic evolution of healthcare utilization and costs from month to month relative to the diagnosis month’s values using an expanded linear regression model by 5 COVID-19 diagnosis month lead and 6 lag terms. The COVID-19 diagnosis month corresponds to the 30 days immediately following a patient’s diagnosis and therefore the post diagnosis period now spans 31–180 days following the COVID-19 diagnosis date.

Finally, we use the healthcare utilization and cost monthly estimates to compute, for each corresponding month-pair before and after diagnosis, excess utilization and excess cost. These excess utilization and cost estimates reflect the difference in outcomes between a given post-COVID-19 diagnosis month and its corresponding pre-diagnosis month. It should be noted that this definition of excess healthcare utilization (and subsequently excess costs) only loosely follows the excess mortality terminology of the CDC defined “as the difference between the observed numbers of deaths in specific time periods and expected numbers of deaths in the same time periods reported by the jurisdiction in which the death occurred”.^1^ Instead, we do not make predictions about the post-diagnosis trajectories of the outcomes but generate reliable evidence based on observed month-to-month differences centered around the diagnosis month as opposed to aggregate-level forecasts based on jurisdiction-specific information.

### Ethics approval

This study was deemed “not regulated” by the University of Michigan Institutional Review Board (HUM00186569).

## Results

### Patient characteristics

Table 1 below summarizes the distribution of the outcome variables before and after COVID-19 diagnosis on aggregate and by CPT code category. The increase in both utilization and costs post-diagnosis is apparent and highlights the general direction of the influence COVID-19 had on these outcomes.

**Table 1:** Summary statistics

**Panel A: Healthcare Utilization (in # of recorded services)**				
Statistic	Mean		Std. Dev	
Time	Pre-diagnosis	Post-diagnosis	Pre-diagnosis	Post-diagnosis
CPT Categories				
All	0.4783	1.2078	4.0839	8.4962
Cardiovascular	0.0345	0.0512	0.3249	0.4209
Emergency/critical care	0.0423	0.1052	0.4865	1.3298
Immunization	0.0053	0.0146	0.1203	0.1770
New patient visit	0.0189	0.0211	0.1583	0.1684
Established patient visit	0.1177	0.1994	0.4857	0.6661
Inpatient	0.2350	0.7688	3.6240	7.5812
Preventive	0.0006	0.0012	0.0247	0.0343
Surgery	0.0153	0.0333	0.2516	0.4322
Urgent Care	0.0069	0.0096	0.1412	0.1796
Telemedicine	0.0346	0.0706	0.2461	0.3896
**Panel B: Medical Expenditures (in $)**				
Statistic	Mean		Std. Dev	
Time	Pre-diagnosis	Post-diagnosis	Pre-diagnosis	Post-diagnosis
CPT Categories				
All	128.0586	351.6679	1182.7833	2473.6259
Cardiovascular	5.2997	8.7476	88.2783	113.4098
Emergency/critical care	34.2311	75.2287	373.9894	923.6099
Immunization	0.5148	1.2295	15.0977	21.0416
New patient visit	5.9323	9.9596	73.0581	164.8569
Established patient visit	21.2023	62.5462	111.3244	577.1386
Inpatient	48.9996	144.7522	802.1973	1409.7630
Preventive	0.1563	0.2934	6.7641	9.1265
Surgery	10.0377	43.3060	340.7676	927.7234
Urgent care	0.3379	0.5380	9.1944	12.2543
Telemedicine	6.3234	13.2536	52.3640	87.3871

Source: COVID-19 Research Database

### Baseline results

**Fig. 2:**
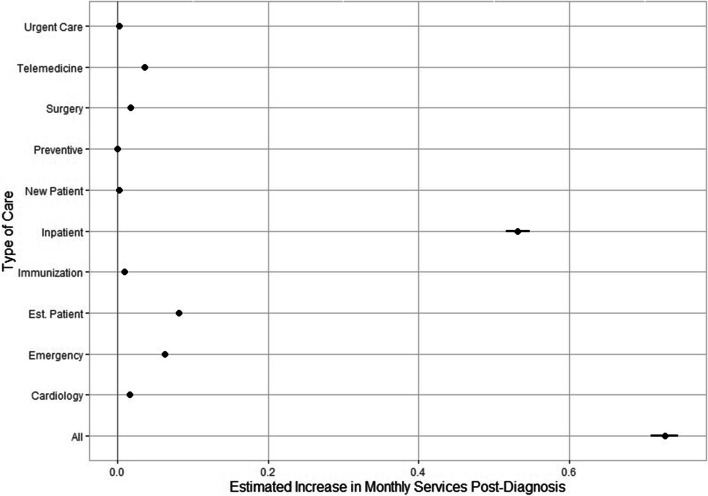
Association of COVID-19 diagnosis with healthcare utilization by type of care. Figure 2 shows the estimated increase in the monthly number of aggregate services and the increases for ten service-specific categories over six months, inclusive of the first 30 days. Each dot on the plot represents the coefficient estimate, the lines extending from each dot represent a 95% confidence interval for each estimate

Next, we present and interpret results based on the baseline model as well as the month-by-month regression analysis. Figure 2 depicts the rise in the monthly number of aggregate services for ten service-specific categories over six months, inclusive of the first 30 days and Additional File 2 contains the corresponding point estimates. COVID-19 diagnosis is associated with 0.7269 (95% CI, 0.7088 to 0.7449 visits; *P* < 0.001) more healthcare visits per month, on aggregate, during a six-month period following COVID-19 diagnosis. The sharpest rise in utilization is reported for inpatient visits (0.5319 more visits, 95% CI 0.5158 to 0.5480 visits; *P* < 0.001) followed by emergency care (0.0626 more visits, 95% CI 0.0600 to 0.0653 visits; *P* < 0.001) and telemedicine (0.0359 more visits, 95% CI 0.0351 to 0.0367 visits; *P* < 0.001). Non-negligible increases are reported for surgery (0.0179 more visits, 95% CI 0.0170 to 0.0190 visits; *P* < 0.001) and cardiology services (0.0165 more visits, 95% CI 0.0156 to 0.0174 visits; *P* < 0.001). On the contrary, marginally more visits for urgent care or by new patients are found and, even smaller increases are estimated for preventive care services. Older adult patients are associated with increasingly more healthcare utilization relative to minors for all categories of services but preventive care post-diagnosis. Males are correlated with greater use of cardiological, emergency or inpatient services post-diagnosis relative to females and lesser immunizations, telemedicine, preventive care, urgent care, new or established patient visits. Relative to the commercially insured, Medicare beneficiaries record more healthcare visits, whereas Medicaid beneficiaries and the uninsured record fewer ones. Patients residing in 3-digit zip codes which are rural are estimated to utilize less healthcare services for all categories but immunization. Those living in areas with an increased share of non-white population record use narrowly more services, albeit the evidence across different types of care is mixed with an increased number of immunizations, inpatient and established patient visits but marginally fewer urgent care and telemedicine visits. We find that patients in 3-digit zip codes of increased per capita income report fewer healthcare visits across all categories. Those living in areas with an increased fraction of the population over 65 years of age utilize fewer services but immunization and established patient visits while those in areas with a larger share of individuals under 18 years old are associated with less healthcare utilization for all categories but established patient visits. Patients in 3-digit zip codes with a relatively high percentage of the population not employed are correlated with increased levels of utilization of all services examined but immunization. Mixed evidence is found regarding the relationship of an increased proportion of female population, population living in poverty or total population and healthcare utilization.

**Fig. 3:**
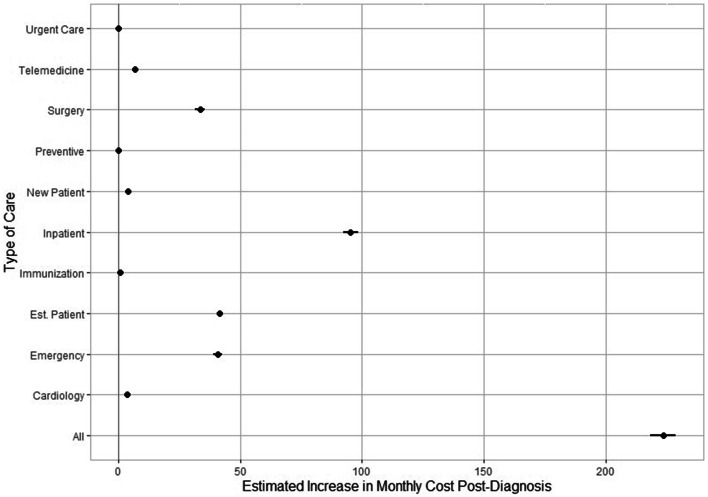
Association of COVID-19 diagnosis with healthcare cost by type of care. Figure 3 depicts the estimated post-diagnosis increase in aggregate monthly healthcare costs and separately for each of the 10 categories examined. Each dot on the plot represents the coefficient estimate, the lines extending from each dot represent a 95% confidence interval for each estimate

Figure 3 depicts the estimated post-diagnosis increase in aggregate monthly healthcare costs and separately for each of the 10 categories examined. We estimate that COVID-19 diagnosis is correlated with an additional $223.59 (95% CI, 218.34 to 228.83; *P* < 0.001) in total monthly medical expenditures, on average, as shown in Additional File 3. This increase was driven primarily by financial costs associated with inpatient care ($95.29, 95% CI $92.20 to $98.39; *P* < 0.001), emergency care ($40.82, 95% CI $39.01 to $42.64; *P* < 0.001) and surgical services ($33.52, 95% CI $31.65 to $35.40; *P* < 0.001)*.* We find smaller but notable increases in medical expenditures for care delivered via telemedicine ($6.90, 95% CI $6.72 to $7.09; *P* < 0.001) and for cardiology services ($3.43, 95% CI $3.18 to $3.67; *P* < 0.001). COVID-19 diagnosis is associated with only marginally higher medical expenditures on preventive care services, urgent care, and immunizations.

Our estimates indicate that males are associated with increased overall medical expenditures relative to females which masks significant heterogeneity as the former use more cardiology and emergency care but record fewer immunizations, telemedicine, preventive care, urgent care, new or established patient visits. Medicare beneficiaries are associated with the largest increase in overall medical expenditures relative to those using private health insurance whereas Medicaid insurance status is associated with a small increase in costs and a reduction for the uninsured. Medical expenditures follow a similar pattern to healthcare utilization as older adults are recording increasingly higher costs relative to minors. There is evidence that 3-digit zip code-level characteristics such as the proportion of the population living in rural areas or per capita income are correlated with reduced medical expenditures across all categories of care. On the contrary, the negative association between total medical expenditures and the poverty rate, total population levels, the proportion of the population that is non-white, 65 years or older and 18 years or younger conceals both positive and negative relationship among different categories of services. Similarly, although the proportion of the female population is not statistically associated with total medical expenditures, it is correlated with non-negligible reductions in emergency care costs as well as minor increases in surgery costs. Also, our estimates suggest that an increase in the share of the population that is not working where patients reside is associated with higher medical expenditures, primarily driven by increases in costs for emergency and inpatient care.

### 30-Day trends of healthcare utilization and medical expenditures

**Fig. 4:**
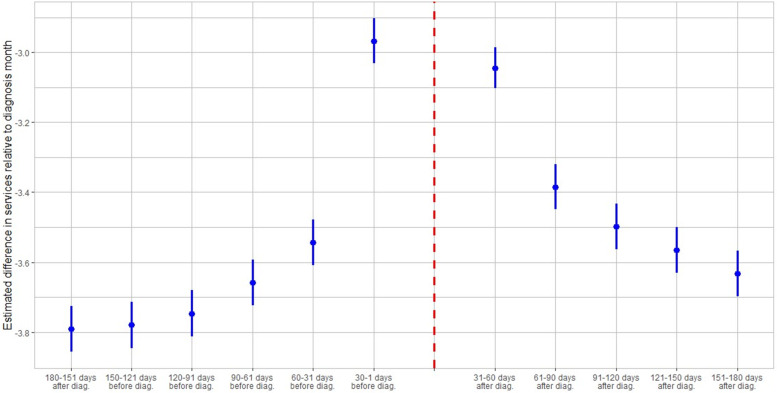
Evolution of healthcare utilization relative to diagnosis month. Figure 4 shows the evolution of healthcare utilization relative to the diagnosis month. Each blue dot on the grid represents the estimated average change in number of monthly services relative to the diagnosis month, in which an average of 4.06 monthly services was recorded. Each blue dot represents a coefficient estimate and the vertical lines are confidence intervals at the 95% level

**Fig. 5:**
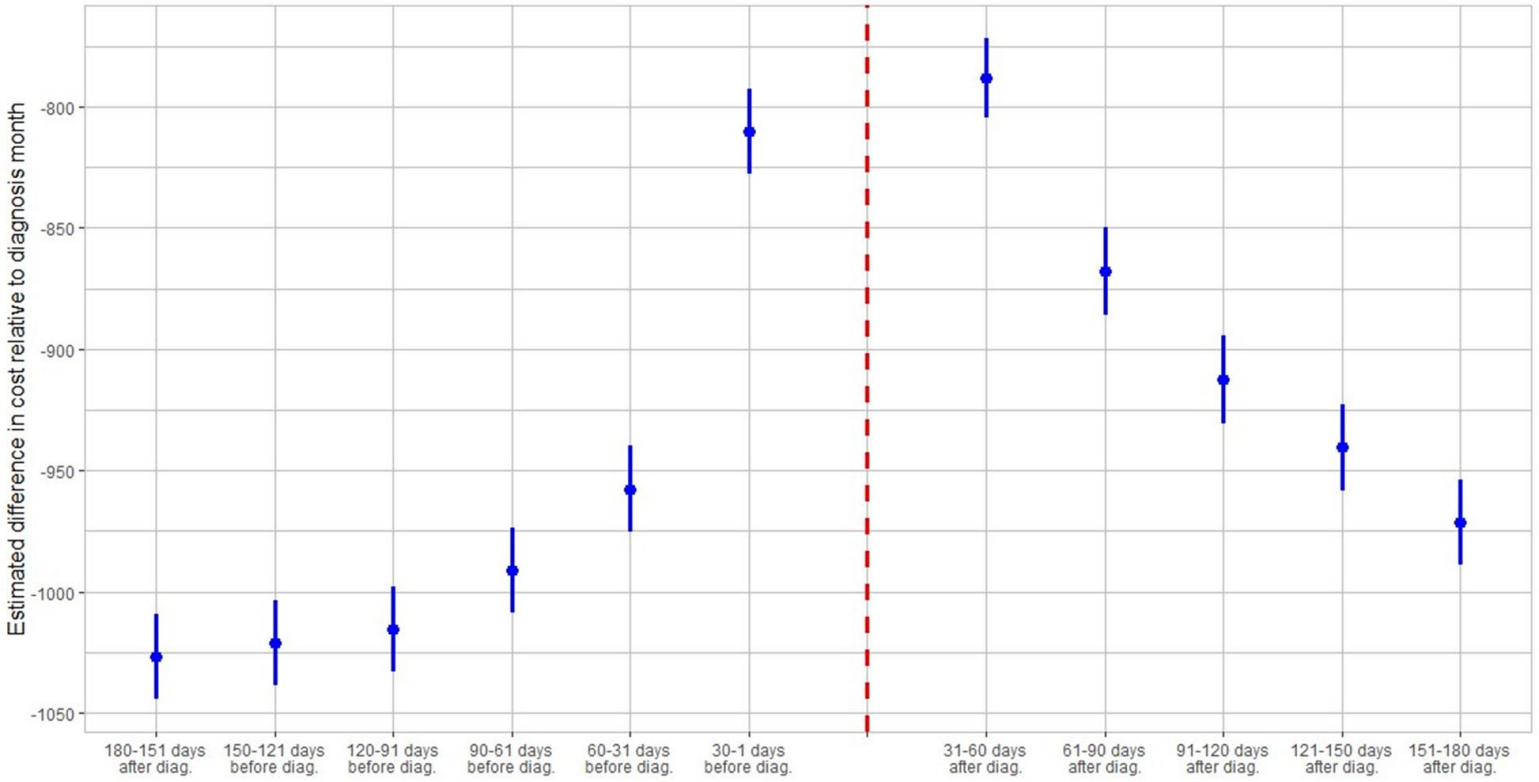
Evolution of healthcare cost relative to diagnosis month. Figure 5 shows the evolution of healthcare cost relative to the diagnosis month. Each blue dot on the grid represents the estimated average change in the monthly cost relative to the diagnosis month, in which an average cost of 1099.49 was recorded. Each blue dot represents a coefficient estimate and the vertical lines are confidence intervals at the 95% level

Figures 4 and 5 display the evolution of utilization of care and medical expenditures of COVID-19 patients from 180 days before a patient-specific diagnosis to 180 days after, in 30-day increments.

Figure 4 shows the progression of healthcare utilization relative to the diagnosis month, adjusted by the explanatory variables used in Eq. (1). Overall healthcare utilization peaked at 4.06 visits in the COVID-19 diagnosis month, sharply rising from pre-diagnosis levels, only to gradually decline during the next five months without reverting to its pre-diagnosis healthcare utilization levels among the general population. Figure 4 also shows that 3.045 fewer services are used in month t + 1 or 31–60 after diagnosis whereas 60–31 days before diagnosis (month t-2) there are 3.543 fewer visits relative to diagnosis month utilization. This amounts to approximately 0.499 more monthly visits in month t + 1 relative to month t-2, on average. Repeating these calculations for subsequent month-pairs, we obtain 0.274 excess relative utilization in the next month-pair [t-3, t + 2], 0.248 excess visits in month-pair [t-4, t + 3], 0.215 excess visits in month-pair [t-5, t + 4], and 0.158 excess visits for the last month-pair [t-6, t + 5]. There is a lead-up of healthcare utilization to the diagnosis date as illustrated by the relatively high utilization levels 30–1 days before diagnosis. This may be attributed to healthcare visits only days prior to the lab-confirmed infection to assess symptoms before the manifestation or clinical detection of COVID-19. The month-specific comparisons imply that the positive baseline estimate is not solely driven by excess use in the COVID-19 diagnosis month but also by utilization and costs in the post-diagnosis months. These findings suggest that COVID-19 patients demand monthly healthcare services at an increased rate for at least six months following diagnosis, on average.

Figure 5 shows healthcare cost trends relative to the diagnosis month, adjusted by the full set of regressors used in Eq. (1). Total monthly medical expenditures attain their highest value at $1099.49 during the diagnosis month. Thereafter, they gradually decline, although less rapidly than healthcare utilization does, and do not revert to their corresponding pre-diagnosis monthly levels. Again, there is evidence of a lead-up of medical expenditures to the diagnosis which can be similarly explained by healthcare visits to address pre-COVID-19 symptoms. Carrying out similar month-pair comparisons uncovers excess relative costs of $169.34, 31–60 days before and after diagnosis to $123.32 in the subsequent month-pair [t-3, t + 2], $102.74 for month-pair [t-4, t + 3], $80.62 for month-pair [t-5, t + 4], and $55.27 for the last month-pair [t-6, t + 5]. These results indicate that COVID-19 patients face persistently higher monthly healthcare costs over a period of six post-diagnosis months relative to pre-diagnosis monthly medical expenditures.

### Heterogeneity analysis

Finally, we explore the heterogeneity of healthcare use and costs in age, using the following four age groupings: individuals 17 years old or younger, 18–44, 45–64, or 65 or more years old.

**Fig. 6:**
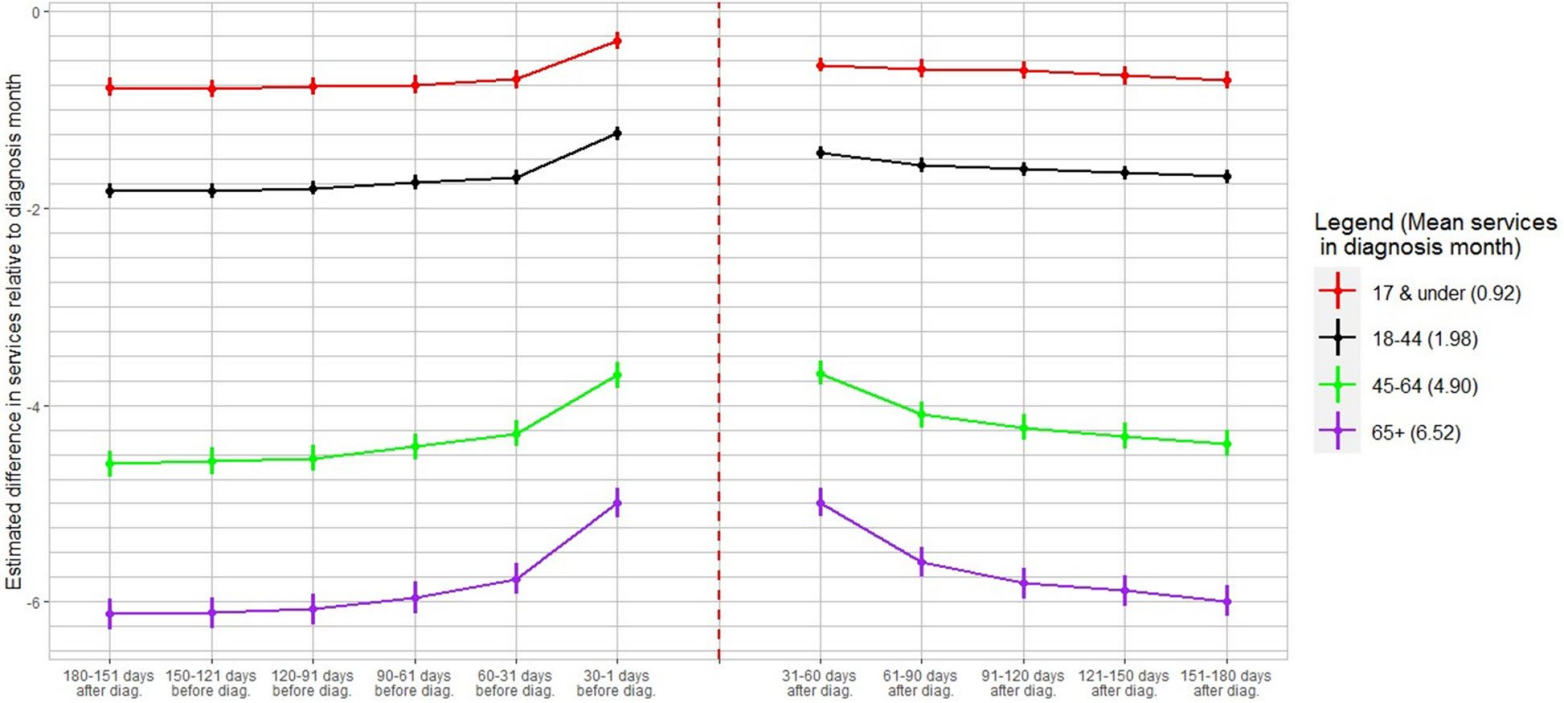
Evolution of healthcare utilization relative to diagnosis month by age. Figure 6 demonstrates the heterogeneity of healthcare utilization across the following seven age groupings: individuals 17 & under, 18–44, 45–64, 40–49, or 65 + years old. The red line depicts the age group 17 & under, which registered an average of 0.92 services in the diagnosis month. The black line depicts the age group 18-44, which registered an average of 1.96 services in the diagnosis month. The green line depicts the age group 45-64, which registered an average of 4.90 services in the diagnosis month. The purple line depicts the age group 65 + , which registered an average of 6.52 services in the diagnosis month. Dots are the coefficient estimates and vertical bars represent confidence intervals at the 95% level

Figure 6 demonstrates that healthcare utilization is greater in the vast majority of post-diagnosis months across all age groups; however, trends are largely heterogeneous. Care sought post-diagnosis is trending downwards but does not vary substantially over time for individuals 17 years old and nearly returns to their pre-diagnosis levels by the sixth month. Similarly, post-diagnosis healthcare utilization by patients who are 18 to 44 years of age exhibits a gentle decline; however, it remains marginally higher than its pre-diagnosis levels throughout the period of analysis. Compared to the younger cohorts, healthcare utilization by the next two cohorts of individuals (45–64 and 65 years of age or older) fluctuates at significantly lower levels relative to diagnosis month use. It steeply drops 31–60 days post-diagnosis to decay at a decreasing rate in subsequent months and, importantly, it does not revert to pre-diagnosis levels. Finally, there is no evidence of a differential lead-up in healthcare utilization 30–1 days prior to diagnosis across age groups; these observations are not used for excess utilization comparisons. Back-of-the-envelope computations shown in Table 2 indicate that excess relative healthcare utilization fluctuated minimally during post-diagnosis months 2–5 for individuals 17 years or younger, ranging between 0.166 and 0.133 visits. By month 6, utilization nears its baseline with only 0.07 more estimated visits relative to its six-month pre-diagnosis levels. On the contrary, we find that excess relative utilization of all adult age groups persisted at higher levels relative to pre-diagnosis months. Among adult groups, the greatest excess healthcare utilization is calculated for the most senior cohort (> 65 years old) in the second and third month away from the diagnosis date with 0.78 and 0.365 excess medical encounters, respectively. Farther out from the diagnosis date though, we compute the highest levels of excess healthcare utilization during the fourth, fifth and sixth months out at 0.312, 0.258 and 0.207 visits, respectively, for patients 55 and 64 years of age. Excess relative utilization of care by individuals 18–44 years old showed resilience post-diagnosis taking values between 0.147 and 0.245 more visits per month.

**Table 2:** Excess monthly healthcare utilization by age category

	(1)	(2)	(3)	(4)	(5)
	All ages	17 & under	18–44	45–64	65 +
31–60 days post vs. 60–31 days pre-	0.499	0.148	0.245	0.612	0.780
61–90 days post vs. 90–61 days pre-	0.274	0.162	0.176	0.328	0.365
91–120 days post vs. 120–91 days pre-	0.248	0.166	0.191	0.312	0.264
121–150 days post vs. 150–121 days pre-	0.215	0.133	0.183	0.258	0.225
151–180 days post vs. 180–151 days pre-	0.158	0.070	0.147	0.207	0.133

*Notes*: Back-of-the envelope calculations based on regression estimates in Additional File 2

**Fig. 7:**
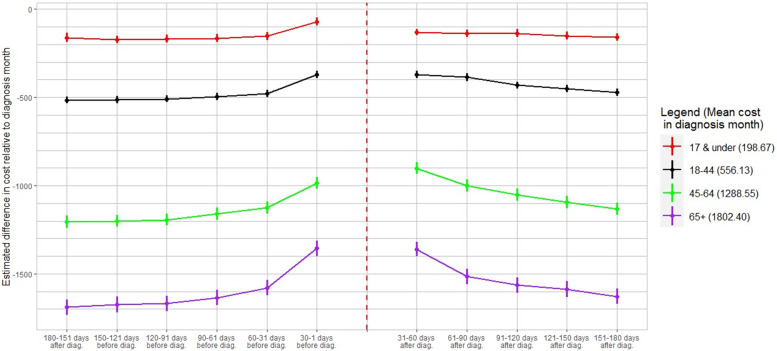
Evolution of healthcare cost relative to diagnosis month by age**.** Figure 7 demonstrates the heterogeneity of healthcare costs across the following seven age groupings: individuals 17 & under, 18–44, 45–64, or 65 + years old. The red line depicts the age group 17 & under, which registered an average cost of 198.67 in the diagnosis month. The black line depicts the age group 18–44, which registered an average cost of 556.13 in the diagnosis month. The green line depicts the age group 45–64, which registered an average cost of 1288.55 in the diagnosis month. The purple line depicts the age group 65 + , which registered an average cost of 1802.40 in the diagnosis month. Dots are the coefficient estimates and vertical bars represent confidence intervals at the 95% level

Figure 7 shows cost estimates for all months relative to the COVID-19 diagnosis month across age groupings. Increased spending is reported for all months following diagnosis across all adult age groups. Whereas patients 17 years old or younger show a virtually flat trend of post-diagnosis utilization that retreats to its pre-diagnosis levels by the sixth month, those 18 to 44 years old exhibit a small, negative linear trend for the duration of the post-diagnosis period. Medical expenditures of individuals 45–64 and 65 years or older decline at a diminishing rate while remaining higher than their pre-diagnosis levels. Back-of-the-envelope calculations presented in Table 3 suggest that excess relative healthcare costs persist for minors for five months, ranging from $22.07 to $31.10 up to five months after diagnosis and, essentially, return to pre-diagnosis levels by the sixth month where only $2.77 in excess costs are reported. However, all adult age groupings exhibit substantially larger excess relative utilization to baseline in all post-diagnosis months. Individuals 18–44 years of age experience substantive excess relative costs from a maximum of $109.64 to a minimum of $44.21 in month six. We find that individuals 45–64 years old experience the largest positive difference in post-diagnosis healthcare costs relative to their pre-diagnosis levels in every post-diagnosis month which range from $222.88 in excess costs 31 to 60 days away from diagnosis to $73.43 in excess costs 151 to 180 days from the diagnosis date. Finally, relatively smaller excess costs are calculated for individuals 65 years old or more which vary from $219.17 in the second month to $60.49 in the sixth month.

**Table 3:** Excess monthly cost by age category

		(1)	(2)	(3)	(4)
	All Ages	17 & under	18–44	45–64	65 +
31–60 days post vs. 60–31 days pre-	169.34	22.89	107.95	222.88	219.17
61–90 days post vs. 90–61 days pre-	123.32	26.02	109.64	159.55	120.30
91–120 days post vs. 120–91 days pre-	102.74	31.10	77.19	142.58	104.49
121–150 days post vs. 150–121 days pre-	80.62	22.07	61.65	107.34	86.80
151–180 days post vs. 180–151 days pre-	55.28	2.77	44.21	73.43	60.49

*Notes*: Back-of-the envelope calculations based on regression estimates in Additional File 3

## Discussion

Using claims data, this study contributes to the long COVID literature by tracking patient outcomes among one of the largest COVID-19 patient cohorts with diverse insurance coverage over a long follow-up period. We compare healthcare utilization and costs of commercially insured, Medicare and Medicaid beneficiaries and uninsured patients within 180 days before and after a lab-confirmed COVID-19 diagnosis during February 2020-October 1, 2020. We estimate that COVID-19 diagnosis is associated with 0.7269 more visits and $223.60 more in healthcare costs per month over a 6-month post-diagnosis period which are comparable in magnitude to evidence from a sample of patients who did not require hospitalization but lower in magnitude than estimates based on hospitalized patients [23–25]. This is may be reflective of our study sample which includes both inpatient and outpatient visits but excludes patients with severe complications of acute inpatient care as their last recorded visit, thereby, reducing the number of higher acuity cases. Furthermore, one should interpret the estimated magnitudes as a conservative estimate of aggregate post-COVID-19 monthly healthcare utilization and medical expenditure levels given our focus on non-pulmonary medical encounters. Compared to other research following COVID-19 patients over the same time duration, we similarly find that healthcare utilization peaks during the first 30 days following diagnosis and steadily decreases over the next five months but lower utilization among females [23]. The discrepancy in findings by gender in most service categories across the two studies could be due to their different geographical settings. Huang et al. (2022) relies on patient data from Southern California, an urban setting in a state with Medicaid availability and, therefore, access to privately- and publicly provided care is relatively broader for females than in the nationwide sample we employ.

The largest increases in utilization are reported for inpatient, emergency care and telemedicine services whereas the narrowest ones for preventive care services, as noted in previous work highlighting deferral of care and the increased use of telemedicine during the COVID-19 pandemic [5, 8, 27, 28]. Inpatient and emergency care and surgery are the categories with the largest excess relative costs, in line with other evidence of the relative contribution of inpatient care in medical expenditures of COVID-19 patients [24]. Notable increases in utilization are reported for established patients but not new patients which is illustrative of difficulties in accessing care among COVID-19 patients requiring new consultations as suggested in other studies [29].

There is corroborating evidence of significant heterogeneity in the post-diagnosis trajectories of COVID-19 patient outcomes by gender and age [22, 23]. Males utilize more healthcare services, on average, although females are associated with increased use of immunization, preventive and urgent care services which reveals differential care needs of COVID-19 patients by gender. Individuals 17 years or younger engage in minor excess relative use and costs which persist for five months. Modest excess relative utilization and costs are reported for the 18–44 age group while estimates are increasing in magnitude for patients 45–64 and those over 65 years of age. The former age cohort is experiencing the largest excess relative utilization in the second and third month before and after diagnosis. However, in subsequent months, patients 45–64 years are recording the highest excess health usage while sustaining the largest excess costs among all age groups in every month. COVID-19 diagnosis is associated with more healthcare visits and greater costs with the latter diminishing more gradually than the former over time. Taken together, this implies that the rise in utilization and costs following COVID-19 diagnosis is manifesting in the form of increasingly costly medical encounters. These reported excesses are rather modest, though, which may be attributed to the exclusion of pulmonary claims from the empirical analysis and increasing baseline, pre-diagnosis usage and cost levels in cohort age. It is reasonable to expect the most senior group to have the highest demand for healthcare services due to their relatively elevated healthcare needs regardless of COVID-19 diagnosis, explaining how the largest magnitudes of COVID-19 diagnosis association with healthcare utilization (only after the third month post-diagnosis) and medical expenditures are rather reported for the second most-senior age cohort.

Our results portray the importance of local-level socio-economic factors in healthcare use and costs such as a negative association with 3-digit zip code-level per capita income which is in line with evidence that individual residing in neighborhoods with greater social vulnerability are more likely to receive high acuity care [30]. Our estimates also confirm that insurance status has been a major determinant of healthcare use and costs during the pandemic. In interpreting differences in observed costs, it is important to recall that medical expenditures can be driven by a combination of prices and utilization. The relatively higher levels of healthcare utilization and expenditures by Medicare beneficiaries to privately insured patients may be explained by the greater disease severity experienced by the relatively older Medicare beneficiaries. Medicaid beneficiaries are correlated with lower costs relative to privately-insured patients despite no significant differences in utilization which may be explained by reimbursementexplained reimbursement differences between Medicaid and commercial insurance as the latter traditionally offers more generous reimbursement rates to providers. This finding is in line with nationwide estimates using claims data suggesting prices paid to hospitals by private insurance increased from 236 percent of Medicare reimbursement rates in 2015 to 241 percent in 2017 [31].

In March 2020, costs of COVID-19 testing and treatment for those who are uninsured became reimbursable at Medicare rates by the HRSA using funding that relies on periodic cash injections by Congress [32]. As expected by the smaller menu of services available, utilization by the uninsured is lower than healthcare use of privately insured patients. However, uninsured patients are associated with relatively increased medical expenditures, driven costs for surgical services, which is suggestive of a relatively costlier utilization bundle. Thus, policymakers should be aware that withdrawing HRSA funding for coverage of COVID-19 related expenses of the uninsured may place a relatively high financial burden on this vulnerable population.

### Limitations

This work is subject to a number of limitations which illustrate the importance of future research on long COVID. First, in the absence of a case–control study design, our results do not have a causal interpretation. Given pre-existing evidence from comparative evaluations of individuals hospitalized with COVID-19 relative to individuals with no evidence of COVID-19 infection or individuals hospitalized with seasonal influenza, this longitudinal analysis instead emphasizes short- and long-term trends of utilization and costs in both inpatient and outpatient settings [12, 33]. Our study design reflects our confidence in these descriptive estimates relative to causal claims which would have been more challenging to verify given the non-random nature of COVID-19 infection and absence of information on patient occupation, educational status and other social determinants of COVID-19 exposure. Also, since the source of our data is on the provider side, we do not have comprehensive data on the health care utilization of patients in our study sample. Therefore, our estimates are a lower bound of health care utilization and costs while there is no reason to believe that the extent of underestimation is different before and after COVID diagnosis. Additionally, our baseline regression models cannot detect whether health care utilization after diagnosis is a result of post COVID-19 symptoms or whether it is due to pre-existing health conditions unrelated to COVID-19. In response, we complement our estimates with excess healthcare utilization and costs measures which, implicitly, capture the presence of pre-existing conditions over a six-month period before COVID-19 diagnosis by netting out pre-diagnosis outcome levels, month-by-month. Moreover, as we do not observe patient mortality, sample attrition may be especially pronounced among older population groups. To address this limitation, we exclude likely non-survivors from the study sample. This implies our estimates are even less likely to overestimate the influence of COVID-19 diagnosis on utilization and costs. The analysis does not consider utilization of pulmonology services despite the direct clinical link between COVID-19 and pulmonary symptoms because our goal is to shed light on the association of COVID-19 diagnosis with utilization and costs associated with COVID-19 diagnosis over a wide variety of non-respiratory related symptoms where an obvious relationship may have not been anticipated. Finally, we do not distinguish among COVID-19 vaccination status given that all individuals in the sample were diagnosed by October 1, 2020, prior to any emergency authorization of COVID-19 vaccines in the US.

## Conclusions

This study provides an overview of healthcare utilization and medical expenditures of COVID-19 patients over a six-month period. Our results should be interpreted as descriptive evidence that excess healthcare utilization and costs of adults persisted for six months following COVID-19 whereas excess use and costs subside after five months for individuals 17 years or younger to their pre-diagnosis levels, on average. Patients over 65 years of age are experiencing the largest post-diagnosis healthcare utilization in the short-term but those between 45–65 years of age record the most elevated excess use in the long term and highest excess medical expenditures. Future studies of utilization and costs of adult COVID-19 patients should employ a longer follow-up period to identify when outcome levels diminish to their pre-diagnosis levels.

Our empirical findings regarding the uninsured have important policy implications by highlighting the important role that emergency HRSA COVID-19 testing and treatment funding has played during the public health emergency in providing access to care. More research is needed to disentangle the effects that racial characteristics have on short- and long-term utilization and medical expenditures by specialty and examine which groups may be subject to inequities in access to care. 

## Supplementary Information

**Additional file 1. **Mechanism and empirical model. Discussion of the study mechanism and the empirical model.

**Additional file 2. **Associations of healthcare utilization by CPT category with COVID-19 diagnosis. Linear regression output of estimated association between healthcare utilization and COVID-19 diagnosis.

**Additional file 3. **Associations of medical expenditures by CPT category with COVID-19 diagnosis. Linear regression output of estimated association between medical expenditures and COVID-19 diagnosis.

## Data Availability

The datasets generated and/or analyzed during the current study are not publicly available due to the nature of claims data but are available from the COVID-19 Research Database for free upon approval [https://covid19researchdatabase.org/].

## Abbreviations

COVID-19: Coronavirus Disease 2019
SD: Standard Deviation
CI: Confidence Interval
BMI: Body Mass Index
HRSA: Health Resources and Services Administration
U.S: United States
E/M: Evaluation and Management
CPT: Current Procedural Terminology
ID: Identifier

## Footnotes

1 Excess mortality definition by the CDC: https://www.cdc.gov/nchs/nvss/vsrr/covid19/excess_deaths.htm
