# Supplementary material for: Long-haul COVID: healthcare utilization and medical expenditures 6 months post-diagnosis
Source: BMC Health Serv Res. 2022 Aug 8;22:1010. doi: 10.1186/s12913-022-08387-3 (PMC9358916; doi:10.1186/s12913-022-08387-3)
Supplement: Supplementary file 1 — Additional file 1. Mechanism and empirical model. Discussion of the study mechanism and the empirical model. [file 12913_2022_8387_MOESM1_ESM.docx]

**Additional File 1. Mechanism and Empirical Model**

*Mechanism*

COVID-19 can influence healthcare utilization through two distinct channels - the direct increase in utilization in response to COVID-19 related illness, which could be exasperated by underlying health conditions, and the indirect impact on non COVID-19 related health conditions that are now treated for due to renewed contact with the health care system. Health shocks can serve to draw patients into the health care system who were not previously engaged. At the same time, health care may be deferred to reduce interaction with the health system that may expose individuals to hospital-afflicted COVID-19 infection or, at the onset of the public health emergency, due to lack of access to care as hospitals scrambled to meet the COVID-19 patient load or social distancing measures were in place. Total healthcare spending will mechanically rise from COVID-19 related visits whereas deferral of non-COVID-19 related care, and particularly preventive care, may reduce healthcare costs in the short term. However, if high-value care was also deferred such as preventive care services, the same individuals may be facing even greater medical expenditures in the long term.

*Linear Regression Model*

We compare utilization of health care services and healthcare expenditures before and after COVID diagnosis using a linear regression model. The estimated model is given in equation (1) below.

$Y_{iszt}={a+\rho POST}_{it}{+\beta X}_{iszt}+\sigma_{s}+\tau_{t}+\varepsilon_{iszt}$ (1) ,

where $Y_{iszt}=\{{USE}_{iszt} measured in number of services by CPT category,{COST}_{iszt} in \$\}$.

We regress healthcare utilization and healthcare expenditures $Y_{iszt}$ for patient *i* in state *s* in month *t* (diagnosis month baseline) in zip-code *z* on a month-specific indicator of an individual’s first COVID-19 diagnosis, ${POST}_{it}$, and a number of explanatory variables collected in vector $X_{iszt}$ including individual-level demographic information such as an individual’s age (grouped in 4 categories), gender, and indicators of the COVID-19 diagnosis month of the year to capture month-specific trends in the evolution of the pandemic as well as aggregate-level socio-economic characteristics such as per capita income, poverty rate, labor force participation rate, total population, the percent of the population that resides in rural areas, is non-white, female, 65 years or older, 18 years or younger at the 3-digit zip code level. This vector also includes three individual insurance status indicators to identify an individual as a Medicare or Medicaid recipient or as uninsured in a given month. We use state fixed effects $\sigma_{s}$ to account for state-invariant factors that may influence our outcomes and cluster standard errors at the individual level. The parameter of interest is *ρ*, the coefficient estimate of ${POST}_{it}$, which measures changes in healthcare utilization and costs six months after a COVID-19 diagnosis relative to their baseline levels during a period of six month periods leading up to the COVID-19 diagnosis date.
