# Supplementary material for: Long-haul COVID: healthcare utilization and medical expenditures 6 months post-diagnosis
Source: BMC Health Serv Res. 2022 Aug 8;22:1010. doi: 10.1186/s12913-022-08387-3 (PMC9358916; doi:10.1186/s12913-022-08387-3)
Supplement: Supplementary file 2 — Additional file 2. Associations of healthcare utilization by CPT category with COVID-19 diagnosis. Linear regression output of estimated association between healthcare utilization and COVID-19 diagnosis. [file 12913_2022_8387_MOESM2_ESM.docx]

**Additional File 2. Associations of Healthcare Utilization by CPT category with COVID-19 Diagnosis**

The first column in Table A.1 shows the coefficient estimate of the COVID-diagnosis indicator and is interpreted as the total increase in healthcare utilization by an individual after her first diagnosis, on average. In columns 2-11, we decompose these aggregate figures into a set of different healthcare services which, respectively, represent cardiology, emergency, immunization, new patient, established patient, inpatient, preventive, surgical, urgent care and telemedicine services.

| Table A.1: Linear Regression Estimates of Monthly Healthcare Utilization Associations with COVID-19 Diagnosis | | | | | | |
| --- | --- | --- | --- | --- | --- | --- |
|  | (1) | (2) | (3) | (4) | (5) | (6) |
|  |  |  |  |  |  |  |
| VARIABLE | All | Cardiology | Emergency | Immunization | New patient | Est. patient |
| COVID-19 Diagnosis | 0.7269*** (0.0092) | 0.0165*** (0.0005) | 0.0626*** (0.0013) | 0.0093***  (0.0002) | 0.0021*** (0.0002) | 0.0814*** (0.0007) |
| Male | 0.2006*** (0.0123) | 0.0085*** (0.0007) | 0.0263*** (0.0016) | -0.0010*** (0.0002) | -0.0025*** (0.0002) | -0.0390*** (0.0013) |
| 18-44 years old | 0.2098*** (0.0121) | 0.0178*** (0.0007) | 0.0286*** (0.0014) | -0.0577*** (0.0014) | 0.0085*** (0.0004) | 0.0130*** (0.0022) |
| 45-64 years old | 0.7517*** (0.0161) | 0.0454*** (0.0009) | 0.0667*** (0.0019) | -0.0557*** (0.0014) | 0.0106*** (0.0004) | 0.0617*** (0.0023) |
| > 65 years old | 0.8973*** (0.0221) | 0.0676*** (0.0013) | 0.0839*** (0.0030) | -0.0546*** (0.0014) | 0.0089*** (0.0004) | 0.0510*** (0.0027) |
| % Rural | -0.0118*** (0.0009) | -0.0008*** (5.65e-5) | -0.0011*** (0.0001) | -1.56e-7  (1.68e-5) | -0.0004*** (1.59e-5) | -0.0011*** (0.0001) |
| % Non-white | 0.0046*** (0.0006) | -3.21e-5 (3.37e-5) | -5.14e-5 (7.81e-5) | 5.24e-5*** (1.01e-5) | -1.95e-5. (1.16e-5) | 0.0004*** (6.5e-5) |
| Per capita income | -1.22e-5*** (9.61e-7) | -5.33e-7*** (5.13e-8) | -8.62e-7*** (1.17e-7) | -1.09e-7*** (1.59e-8) | -1.74e-7*** (1.8e-8) | -8.51e-7*** (1.07e-7) |
| % 65+ | -0.0576*** (0.0038) | -0.0034*** (0.0002) | -0.0060*** (0.0005) | 0.0003*** (6.71e-5) | -1.24e-5 (7.4e-5) | 0.0025*** (0.0004) |
| % <18 years old | -0.0343*** (0.0035) | -0.0016*** (0.0002) | -0.0002 (0.0004) | 1.67e-5  (5.88e-5) | -0.0005*** (5.8e-5) | 0.0015*** (0.0003) |
| Labor force participation rate | 0.0410*** (0.0038) | 0.0026*** (0.0002) | 0.0039*** (0.0005) | -0.0004*** (5.73e-5) | 0.0008*** (5.86e-5) | 0.0015*** (0.0004) |
| % Female | -0.0013 (0.0089) | 0.0015** (0.0005) | -0.0048*** (0.0011) | -0.0004**  (0.0001) | 0.0006** (0.0002) | -0.0081*** (0.0010) |
| Poverty rate | -0.0489*** (0.0021) | -0.0021*** (0.0001) | -0.0038*** (0.0003) | 0.0002*** (3.83e-5) | -0.0005*** (3.57e-5) | 2.91e-5 (0.0002) |
| Total pop. | -6.86e-8*** (8.57e-9) | -2.65e-9*** (4.64e-10) | -9.12e-9*** (1.05e-9) | 5.05e-10*** (1.35e-10) | 6.08e-11 (1.42e-10) | 3.07e-9*** (8.03e-10) |
| Medicare | 0.2828*** (0.0264) | 0.0133*** (0.0016) | 0.0319*** (0.0038) | -0.0039*** (0.0002) | -0.0073*** (0.0004) | -0.0397*** (0.0026) |
| Medicaid | -0.1370*** (0.0202) | -0.0134*** (0.0009) | -0.0009 (0.0028) | -0.0073*** (0.0004) | -0.0158*** (0.0003) | -0.1154*** (0.0018) |
| Uninsured | -0.6937*** (0.0114) | -0.0422*** (0.0006) | -0.0608*** (0.0018) | -0.0042*** (0.0002) | -0.0054*** (0.0004) | -0.0924*** (0.0015) |
|  |  |  |  |  |  |  |
| R-squared | 0.01284 | 0.00994 | 0.00459 | 0.01086 | 0.00563 | 0.02723 |
| Observations | 3,006,168 | 3,006,168 | 3,006,168 | 3,006,168 | 3,006,168 | 3,006,168 |
|  |  |  |  |  |  |  |
|  | (7) | (8) | (9) | (10) | (11) |  |
| VARIABLE | Inpatient | Preventive | Surgery | Urgent care | Telemedicine |  |
| COVID-19 Diagnosis | 0.5319*** (0.0082) | 0.0006*** (3.34e-5) | 0.0179*** (0.0005) | 0.0027***  (0.0002) | 0.0359*** (0.0004) |  |
| Male | 0.2078*** (0.0107) | -0.0002*** (3.89e-5) | 0.0011 (0.0007) | -0.0015*** (0.0002) | -0.0137*** (0.0006) |  |
| 18-44 years old | 0.1977*** (0.0104) | -0.0056*** (0.0002) | 0.0032*** (0.0005) | 0.0054***  (0.0005) | 0.0054*** (0.0010) |  |
| 45-64 years old | 0.6063*** (0.0141) | -0.0057*** (0.0002) | 0.0201*** (0.0009) | 0.0013**  (0.0004) | 0.0188*** (0.0011) |  |
| > 65 years old | 0.7207*** (0.0191) | -0.0060*** (0.0002) | 0.0274*** (0.0012) | -0.0028*** (0.0004) | 0.0057*** (0.0012) |  |
| % Rural | -0.0078*** (0.0007) | 1.61e-5*** (3.4e-6) | -0.0005*** (4.27e-5) | -0.0002*** (1.6e-5) | -0.0010*** (4.38e-5) |  |
| % Non-white | 0.0045*** (0.0006) | -1.81e-6 (1.74e-6) | 6.95e-6 (3.84e-5) | -0.0001*** (1.5e-5) | -0.0003*** (3.02e-5) |  |
| Per capita income | -8.95e-6*** (8.41e-7) | -1.78e-8*** (2.56e-9) | -4.76e-7*** (5.64e-8) | -8.86e-8*** (2.01e-8) | -6.54e-7*** (4.71e-8) |  |
| % > 65 years old | -0.0480*** (0.0033) | -3.7e-5*** (1.06e-5) | -0.0022*** (0.0002) | -0.0003*** (6.3e-5) | 1.08e-5 (0.0002) |  |
| % <18 years old | -0.0305*** (0.0031) | -2.45e-6 (1.22e-5) | -0.0020*** (0.0002) | -0.0006*** (6.2e-5) | -0.0011*** (0.0002) |  |
| Labor force participation rate | 0.0308*** (0.0033) | -0.0001*** (1.23e-5) | 0.0014*** (0.0002) | 0.0007*** (5.66e-5) | 0.0022*** (0.0002) |  |
| % Female | 0.0037 (0.0078) | -5.87e-5* (2.36e-5) | 0.0035*** (0.0006) | 0.0034***  (0.0002) | -0.0034*** (0.0005) |  |
| Poverty rate | -0.0402*** (0.0019) | 6.53e-5*** (6.72e-6) | -0.0017*** (0.0001) | -0.0006*** (3.74e-5) | -0.0005*** (9.82e-5) |  |
| Total pop. | -5.76e-8*** (7.48e-9) | 1.6e-10*** (2.65e-11) | -2.16e-9*** (5.1e-10) | -1.29e-10 (1.49e-10) | 1.14e-9** (3.88e-10) |  |
| Medicare | 0.2667*** (0.0230) | -0.0004*** (2.53e-5) | 0.0242*** (0.0015) | -0.0042*** (0.0003) | -0.0227*** (0.0011) |  |
| Medicaid | 0.0344. (0.0179) | -0.0011*** (6.7e-5) | -0.0068*** (0.0008) | -0.0090*** (0.0002) | -0.0502*** (0.0008) |  |
| Uninsured | -0.5129*** (0.0098) | -0.0010*** (3.25e-5) | 0.0378*** (0.0017) | -0.0117*** (0.0003) | -0.0602*** (0.0008) |  |
|  |  |  |  |  |  |  |
| R-squared | 0.00879 | 0.00314 | 0.00622 | 0.00249 | 0.01501 |  |
| Observations | 3,006,168 | 3,006,168 | 3,006,168 | 3,006,168 | 3,006,168 |  |
| *Notes*: Coefficient estimates represent output of aggregate or care-specific linear regressions. The coefficient on COVID-19 Diagnosis denotes the estimated increase in service utilization after COVID-19 diagnosis. Results are adjusted by gender, 4 age categories (<18 years as the reference category, 18-44, 45-64, and 65+), insurance status (private insurance as reference category, Medicare, Medicaid, uninsured), socioeconomic factors at the 3-digit zip code level, month of COVID-19 diagnosis indicators and state fixed effects. *** p<0.01, ** p<0.05, * p<0.1 | | | | | | |
