# Supplementary material for: Long-haul COVID: healthcare utilization and medical expenditures 6 months post-diagnosis
Source: BMC Health Serv Res. 2022 Aug 8;22:1010. doi: 10.1186/s12913-022-08387-3 (PMC9358916; doi:10.1186/s12913-022-08387-3)
Supplement: Supplementary file 3 — Additional file 3. Associations of medical expenditures by CPT category with COVID-19 diagnosis. Linear regression output of estimated association between medical expenditures and COVID-19 diagnosis. [file 12913_2022_8387_MOESM3_ESM.docx]

**Additional File 3. Associations of Medical Expenditures by CPT category with COVID-19 Diagnosis**

Column (1) of Table A.2 below denotes the point estimate of the diagnosis indicator on medical expenditures and reflects the total increase in healthcare costs by an individual after her first COVID-19 diagnosis, on average. Again, the estimates of cardiology, emergency, immunization, new patient, established patient, inpatient, preventive, surgical, urgent care and telemedicine services related costs following COVID-19 diagnosis are shown in columns 2-11.

| Table A.2: Linear Regression Estimates of Monthly Medical Expenditures with COVID-19 Diagnosis | | | | | | |
| --- | --- | --- | --- | --- | --- | --- |
|  | (1) | (2) | (3) | (4) | (5) | (6) |
|  |  |  |  |  |  |  |
| VARIABLE | All | Cardiology | Emergency | Immunization | New patient | Est. patient |
| COVID-19 Diagnosis | 223.6*** (2.677) | 3.426*** (0.1240) | 40.82*** (0.9245) | 0.7143***  (0.0213) | 4.069*** (0.1678) | 41.66*** (0.5607) |
| Male | 40.15*** (3.388) | 1.529*** (0.1521) | 17.13*** (1.147) | -0.0042  (0.0262) | -1.617*** (0.1809) | -10.90*** (0.5963) |
| 18-44 years old | 61.09*** (3.176) | 2.509*** (0.1420) | 24.62*** (1.070) | -4.395***  (0.1496) | 1.733*** (0.1928) | -0.7314 (0.6187) |
| 45-64 years old | 210.4*** (4.072) | 7.583*** (0.1843) | 52.74*** (1.365) | -4.210***  (0.1498) | 4.675*** (0.2157) | 22.42*** (0.7021) |
| > 65 years old | 271.3*** (6.086) | 11.94*** (0.2971) | 64.78*** (2.117) | -4.052***  (0.1515) | 4.432*** (0.2828) | 35.09*** (1.127) |
| % Rural | -2.801*** (0.2237) | -0.1606*** (0.0109) | -0.8302*** (0.0760) | -0.0041*  (0.0019) | -0.0859*** (0.0088) | -0.0778* (0.0321) |
| % Non-white | 0.6227*** (0.1765) | -0.0216** (0.0076) | -0.1995*** (0.0570) | 0.0069***  (0.0012) | 0.0172* (0.0087) | 0.2934*** (0.0305) |
| Per capita income | -0.0033*** (0.0003) | -6.71e-5*** (1.33e-5) | -0.0005*** (8.55e-5) | -4.82e-6*  (2.01e-6) | -0.0001*** (1.35e-5) | -0.0005*** (4.61e-5) |
| % > 65 years old | -17.48*** (1.045) | -0.4520*** (0.0524) | -5.233*** (0.3743) | 0.0661***  (0.0094) | -0.1016* (0.0414) | 0.0695 (0.1429) |
| % <18 years old | -13.40*** (0.9617) | -0.2861*** (0.0425) | -0.9082** (0.3133) | -0.0203**  (0.0063) | -0.4898*** (0.0436) | -2.652*** (0.1495) |
| Labor force participation rate | 10.66*** (0.9855) | 0.4538*** (0.0410) | 3.732*** (0.3754) | -0.0231*** (0.0061) | 0.1035** (0.0393) | 0.0758 (0.1288) |
| % Female | -2.113  (2.481) | -0.0464 (0.1294) | -4.386*** (0.8017) | -0.1031*** (0.0199) | 0.2980** (0.1139) | 0.4614 (0.4167) |
| Poverty rate | -11.20*** (0.5468) | -0.2592*** (0.0247) | -2.744*** (0.1954) | 0.0127**  (0.0041) | -0.1520*** (0.0291) | -0.1013 (0.0960) |
| Total population | -1.95e-5*** (2.44e-6) | -4.05e-7*** (1.06e-7) | -7.62e-6*** (7.55e-7) | 5.19e-8** (1.59e-8) | 2.24e-7. (1.3e-7) | 2.19e-6*** (4.16e-7) |
| Medicare | 102.6*** (7.848) | 1.257** (0.3824) | 20.98*** (2.613) | -0.3664*** (0.0301) | -2.047*** (0.2617) | -17.87*** (1.066) |
| Medicaid | -38.10*** (5.358) | -2.398*** (0.2021) | -6.305** (1.968) | -0.7132*** (0.0389) | -5.120*** (0.1498) | -25.63*** (0.5381) |
| Uninsured | 160.7*** (7.200) | -6.817*** (0.1349) | -46.29*** (1.326) | -0.2436*** (0.0290) | 19.94*** (0.8775) | 138.0*** (2.908) |
|  |  |  |  |  |  |  |
| R-squared | 0.0132 | 0.00388 | 0.00477 | 0.00450 | 0.00463 | 0.01659 |
| Observations | 3,006,168 | 3,006,168 | 3,006,168 | 3,006,168 | 3,006,168 | 3,006,168 |
|  |  |  |  |  |  |  |
|  | (7) | (8) | (9) | (10) | (11) |  |
| VARIABLE | Inpatient | Preventive | Surgery | Urgent care | Telemedicine |  |
| COVID-19 Diagnosis | 95.29*** (1.580) | 0.1364*** (0.0091) | 33.52*** (0.9580) | 0.1659***  (0.0137) | 6.902*** (0.0955) |  |
| Male | 37.12*** (2.046) | -0.0663*** (0.0101) | -3.282** (1.021) | -0.0911*** (0.0174) | -2.374*** (0.1335) |  |
| 18-44 years old | 36.96*** (1.771) | -1.207*** (0.0520) | 1.109  (1.141) | 0.2795***  (0.0309) | 2.073*** (0.2069) |  |
| 45-64 years old | 103.8*** (2.390) | -1.200*** (0.0519) | 21.99*** (1.322) | 0.0334  (0.0297) | 5.035*** (0.2253) |  |
| > 65 years old | 136.5*** (3.698) | -1.312*** (0.0520) | 20.63*** (1.671) | -0.2067*** (0.0302) | 2.308*** (0.2682) |  |
| % Rural | -1.529*** (0.1396) | 0.0048*** (0.0007) | -0.0636 (0.0517) | -0.0141*** (0.0012) | -0.1757*** (0.0098) |  |
| % Non-white | 0.3625*** (0.1084) | -0.0009* (0.0004) | 0.1550** (0.0541) | 0.0010  (0.0010) | -0.0452*** (0.0067) |  |
| Per capita income | -0.0015*** (0.0002) | -4.63e-6*** (7.08e-7) | -0.0005*** (7.83e-5) | -4.5e-6**  (1.54e-6) | -9.01e-5*** (1.12e-5) |  |
| % > 65 years old | -10.62*** (0.6331) | -0.0138*** (0.0026) | -0.8362** (0.2671) | 0.0167***  (0.0048) | -0.0704 (0.0449) |  |
| % <18 years old | -6.040*** (0.5813) | -0.0139*** (0.0028) | -2.505*** (0.2831) | -0.0189*** (0.0040) | -0.3443*** (0.0343) |  |
| Labor force participation rate | 5.400*** (0.5839) | -0.0269*** (0.0031) | 0.7652** (0.2514) | 0.0430***  (0.0033) | 0.3927*** (0.0371) |  |
| % Female | -0.3641 (1.584) | -0.0186** (0.0059) | 2.354*** (0.6534) | 0.0820***  (0.0135) | -0.7348*** (0.1058) |  |
| Poverty rate | -6.423*** (0.3282) | 0.0166*** (0.0017) | -1.269*** (0.1611) | -0.0499*** (0.0028) | -0.0844*** (0.0221) |  |
| Total population | -1.49e-5*** (1.46e-6) | 6.2e-8*** (7.01e-9) | 1.56e-6* (7.65e-7) | 1.69e-8  (9.89e-9) | 2.18e-7* (8.95e-8) |  |
| Medicare | 94.37*** (5.197) | -0.1035*** (0.0073) | 5.383** (1.715) | -0.2336*** (0.0165) | -2.904*** (0.2987) |  |
| Medicaid | 13.49*** (3.543) | -0.3287*** (0.0149) | -9.976*** (0.9249) | -0.4275*** (0.0187) | -8.945*** (0.1845) |  |
| Uninsured | -86.70*** (1.890) | -0.2819*** (0.0096) | 133.3*** (4.472) | -0.5806*** (0.0205) | -11.29*** (0.1553) |  |
|  |  |  |  |  |  |  |
| R-squared | 0.00873 | 0.00228 | 0.00509 | 0.00155 | 0.01049 |  |
| Observations | 3,006,168 | 3,006,168 | 3,006,168 | 3,006,168 | 3,006,168 |  |
| *Notes*: Coefficient estimates represent output of aggregate or care-specific linear regressions. The coefficient on COVID-19 Diagnosis denotes the estimated increase in service utilization after COVID-19 diagnosis. Results are adjusted by gender, 4 age categories (<18 years as the reference category, 18-44, 45-64, and 65+), insurance status (private insurance as reference category, Medicare, Medicaid, uninsured), socioeconomic factors at the 3-digit zip code level, month of COVID-19 diagnosis indicators and state fixed effects. *** p<0.01, ** p<0.05, * p<0.1 | | | | | | |
